# Assessment of the performance of nurses based on the 360-degree model and fuzzy multi-criteria decision-making method (FMCDM) and selecting qualified nurses

**DOI:** 10.1016/j.heliyon.2020.e03257

**Published:** 2020-01-27

**Authors:** Rahati Meghdad, Rohollahi Nayereh, Sakeni Zahra, Zahed Houriye, Nanakar Reza

**Affiliations:** a Health Services Management, Kashan University of Medical Sciences, Kashan, Islamic Republic of Iran; b Health Services Management, Tehran University of Medical Sciences, Tehran, Islamic Republic of Iran; c Kashan University of Medical Sciences, Kashan, Islamic Republic of Iran; d Ministry of Health and Medical Education, Tehran, Islamic Republic of Iran; e Cancer Research Center, Yasrebi Hospital, Kashan, Islamic Republic of Iran

**Keywords:** Health profession, Nursing, Assessing the performance, 360-Degree model, Fuzzy multi-criteria decision-making method and qualified nurses

## Abstract

**Background:**

Human resources is the most prominent asset of an organization. Despite the constant effort to design optimal and effective systems for assessing employees, evidence shows that managers are not satisfied with the methods and systems to assess employees.

**Objectives:**

Researchers wanted to assess the performance of nurses based on the 360-degree model and fuzzy multi-criteria decision-making technique (FMCDM) and selecting qualified nurses.

**Methods:**

The present study is descriptive and conducted in 2016 in a hospital at Kashan University of Medical Sciences. This study conducted in three ‏stages. 1) Identification of criteria and sub-criteria for the performance assessment that classified into five groups (technical skills, human skills, and perceived skills; individual characteristics; and compliance with the organization's rules and regulations)*.* 2) Weighing the criteria and sub-criteria based on the DEMATEL-ANP (DANP) method in a fuzzy environment. 3) Assessing the performance of nurses based on the 360-degree model, which includes supervisors, coworkers, self-assessment, and patients and their companions. In this stage, four groups used the VIKOR questionnaire to assess the performance.

**Results:**

Among five criteria of assessment, “Human Skills” earned a top score, and among 21 sub-criteria, “Identify the strengths and weaknesses,” “Suitable relationships with patients,” and “Partnership with colleagues” earned the top score. In the 360-degree model, the supervisor's assessment score was 0.521, with the highest weight, and the self-assessment was 0.042 with the lowest weight. Finally, nurse 3 in children and infants ward earned the highest ranking.

**Conclusions:**

The advantage of the proposed method is more realistic results than other methods because the criteria and sub-criteria are weighted, and the importance of each is determined. Hospitals can use the results of this study to assess the performance of medical groups.

## 1 Introduction

Human resources is the most prominent asset and cornerstone of the progress of an organization. Development and improvement in human resources have always been the intention of managers to increase productivity [1, 2]. The importance of human resources in health care organizations, especially in hospitals, has doubled. Nurses, as the largest provider of health services, play a vital role in maintaining care and accountability in services [3]. According to management experts, an appropriate way of human resource development is the assessing of performance, which is a critical process, and a most sensitive issue [4]. Performance assessment helps nurses to adapt their practices to professional standards, which results in more specialization and competence [3, 5, 6].

In this regard, organizations need a system for assessing the qualification of employees to use in executive decision-making, growth, and employee development, and research [7, 8, 9, 10]. Currently, different methods of assessment are being designed and used to address the diverse needs of an organization regarding various aspects. These methods include the Balance Score Card (BSC), 360-Degree feedback, Total Quality Management (TQM), European Foundation for Quality Management (EFQM), Malcolm Baldrige National Quality Award (MBNQA), ISO 9000 Quality Management, Data Envelopment Analysis (DEA), Fuzzy Multi-Criteria Decision-Making methods (FMCDM), Benchmarking etc [11].

Despite the constant effort to design optimal and effective systems for assessing employees, Despite the constant effort to design optimal and effective systems for assessing employees, evidence shows that managers are not satisfied with the methods and systems for employee assessment; the main reasons are the complexity of the assessment process and the presence of defects in the comprehensive assessment system [1, 12]. However, studies suggest that the 360-degree model is one of the methods responding to the need of organizations, and many of the world's leading companies and organizations use this method to assess their managers [13, 14, 15, 16, 17]. In the health sector, it has been mentioned as a successful method for many residency programs [18]. Moreover, according to *Fortune* magazine, more than 80% of companies use 360-degree feedback.

Therefore, researchers intended to identify the criteria and sub-criteria of the performance assessment of nurses in a Hospital of Kashan University of Medical Sciences and determine the weight of each criterion and sub-criteria using fuzzy multi-criteria decision-making methods. Finally, based on the 360-degree model, assessment of the performanceof nurses was carried out and qualified nurses were selected.

## 2 Literature review

### 2.1 Performance assessment criteria

Different criteria defined for performance assessment, depending on the type of activities in the organization [19]. Katz considered successful management as being based on having technical, human, and perceived skills [20]. Boyatizis et al. found attributes, skills, social role, self-image, or work-related knowledge to assess motivational criteria [21]. Furnham categorized the components of competence into five groups: professional knowledge, skills, personality traits, professional credit, and general credit [22]. According to Momeni and Jahanbazai, there are two dimensions of assessment: personal (skills, expert knowledge, personality traits, attitudes, and insights) and social (substantial formal and informal connections) [23]. Catlett & Lovan introduced four categories of personal traits and characteristics, technical skills and care management, work environment and colleagues, and care and caring behaviors for assessing the performance of nurses [24]. Smith and Godfree identified personal and professional characteristics, being patient-centered, support, competence, critical thinking, and patient care as criteria for a competent nurse [25].

### 2.2 360-Degree feedback

In addition to the assessment criteria, the selection of assessors is also essential. In most organizations, the direct supervisor or the manager conducts the assessment. Given the complexity of today's jobs, it is unrealistic to assume that one person can thoroughly observe and assess another individual's performance [22]. In this regard, multi-source performance assessment models can be mentioned which were used for the first time in the British Army from 1940 to 1950 and then expanded to the United States. From the 1960s–1970s, this type of assessment system was considered by the American IBM Bank and the Gulf Oil Company for job promotion [26]. Over the past decades, the concept of performance assessment based on multi-source or multi-degree assessment is considered as a 360-degree model [27]. A 360-degree model is a comprehensive and stakeholder-based process that takes place in a group [28]. This assessment is divided into two categories: formative and summative. Formative assessment provides feedback to individuals, whereas management and promotion purposes use summative assessment [15].

### 2.3 Fuzzy multi-criteria decision-making method (FMCDM)

Managers often need to decide about issues that are not one-dimensional. There are various quality and quantity criteria that complicate the decision-making process. Often, it is necessary to select an option among existing options or rank existing options. The wrong decision will reduce the decision maker's credit, and it will increase the costs of the organization. Hence, the decision-maker needs to use valid methods for decision-making. Today, multi-criteria decision-making methods have been widely used in many fields, which is due to the high ability of these methods in modeling issues and simplifying them to facilitate ease of use [29].

1 Network Analysis Process (ANP)

Weighting is one of the essential steps in multi-criteria decision-making. In weighting, respondents determine the importance of criteria. For weighting criteria, multi-criteria decision-making methods are widely used. The analytic hierarchy process (AHP) is one of the most accepted methods for weighting. In AHP, the elements of each level depend solely on higher-level elements; that is, the coefficients of the importance of elements are necessarily determined by the higher level, while there are often differences between decision alternatives and decision-making criteria, relationships and correlation. The analysis network process (ANP) provides a framework for considering the relationship between decision levels and decision criteria. ANP can be used as a utility tool for issues that create network structures. The use of ANP instead of AHP has increased in most scientific fields in recent years. The main advantage of this method is in solving problems with complex relationships [30].

2 DEMATEL

Also, ANP is an appropriate method for examining the internal communication among the criteria that is not complete. Therefore, the decision-making trial and evaluation laboratory method (DEMATEL) is used to make causal relationships among the criteria as an intuitive structural model and as an effective way to manage the interdependence of criteria. DEMATEL is a comprehensive method for designing and analyzing models with a complex causal structure among the criteria. DEMATEL's final product is a visual map in which the relationships among the criteria are displayed and help the manager to solve the problem [31]. DEMATEL does not work independently, but as a subsystem of a more extensive system such as ANP [32].

3 VIKOR

In multi-criteria decision making, there are methods such as VIKOR and TOPSIS for the ranking of options. Depending on the issue, if the goal is to rank the options, it is better to use the TOPSIS method, but if the goal is to choose the best option, the VIKOR method is appropriate. VIKOR is a multi-criteria decision-making method that selects the best option and brings it as close as possible to the ideal option. VIKOR was derived from the Serbian name “VIsekriterijumska optimizacija i KOmpromisno Resenje” which means “Multi-criteria optimization and compromise solution [33, 34]”. This method is suitable for decision making on issues with inappropriate criteria (different measurement units) and those which are conflicting [35].

4 Fuzzy

Decision-makers often face uncertainties in the decision-making process. In other words, the natural language to express perception or judgment, intellectually) is uncertain and ambiguous. The fuzzy theory was presented to solve this problem by Zade in 1965. Fuzzy word is inaccurate, obscure, and vague. Fuzzy logic considers numbers between zero and one and measures the correctness of anything with a number whose value is between zero and one.

In the fuzzy theory Linguistic Variables are used, the values are fuzzy words instead of numbers. Fuzzy words, while inaccurate, are very understandable [36].

## 3 Method

The present study is descriptive and was conducted in 2016 in a Hospital of Kashan University of Medical Sciences as follows:

### 3.1 Stage 1: Identification of Criteria and Sub criteria for Performance Assessment

According to library studies, the performance assessment criteria of nurses was classified into five groups. These criteria include technical skills, human skills, and perceived skills (Katz skills); individual characteristics; and compliance with the hospital's rules and regulations, which are as follows:

1. Technical skills: Knowledge and ability to perform specific tasks (skills in working with equipment and following procedures)
2. Human skills: Ability to work with individuals and groups, influence their perception, and motivate them
3. Conceptual skills: Ability to understand the complexities of the whole organization and understanding of all elements and components of activities as an entire unit
4. Individual characteristics: Individual and personal attributes of the nurses
5. Compliance with the rules and regulations of the hospital: The requirements that employees observe when entering the ward, working in the ward, and leaving the ward.

Based on five performance assessment criteria, 46 sub-criteria were extracted. As the performance assessment criteria vary from one organization to another, depending on the structure, goals, and mission of organization *(this subject has been considered by many experts such as Lia and Parker; Lynch and Cross; Dixon, Kaplan, and Norton; and Fortune and Nili)* and according to the opinion of the professors and experts of the hospital (through interviews), unrelated criteria were removed and similar criteria were combined. Ultimately, 21 sub-criteria of performance assessment were selected.

### 3.2 Stage 2: Weighing the criteria and sub-criteria based on the DEMATEL-ANP (DANP) Method in a Fuzzy Environment

In this study eight experts were asked to make paired comparisons among the criteria and the sub-criteria. Experts' judgments were based on linguistic options and fuzzy positive numbers (Table 1).

**Table 1:** Language variables for ranking options.

Importance	Very Low: Equal importance	Low: A little more importance	Medium: importance	More: More importance	Very much: Absolute importance
Fuzzy Numbers	(1,1,3)	(1,3,5)	(3,5,7)	(5,7,9)	(7,9,9)

In the next step, based on the calculations of the FDEMATEL method, weighing the criteria and the sub-criteria was done by the fuzzy ANP method.

### 3.3 Stage 3 assessing the performance of nurses based on 360-degree model

According to the 360-degree approach, four assessment groups assess the performance of nurses including: Supervisors, co-workers, patients or their companions, and self-assessment.

In this stage, four groups used the VIKOR questionnaire to assess the performance of nurses. The assessment was conducted as follows:

- In an assessment by the supervisor, the supervisor of each ward in the hospital ranks nurses. To collect data, the census method was used, so all nurses were selected for the assessment by their supervisors.
- In an assessment by co-workers, at least two co-workers rank nurses.
- In self-assessment, nurses assess themselves. They ranked themselves based on their perceptions.
- In an assessment by the patients and their companions, interviewed interviews with patients and their companions was carried out. The stratified sampling method was used to collect patient data. To determine the sample size, the Cochran formula with a 5% error was used. A sample size of 360 people was selected from the admitted patients (5760). The share of work and time dedicated to each of the wards were determined according to the hospital wards (8 wards). Patients were selected in a simple random manner so that patients in each ward had an equal opportunity to be chosen.

### 3.4 Concept Model

The process of assessing the performance of nurses based on the 360-degree model and fuzzy multi-criteria decision-making technique (FMCDM) and selection of qualified nurses is as follows:

### 3.5 Ethical considerations

Before conducting the study, the purpose of the study was explained to the staff and patients in the hospital in accordance with their level of knowledge and information was given to them about their right to refuse to participate in the study if they did not wish to. All individuals were assured that the confidentiality of the information they gave would be preserved, as the questionnaires were anonymous.

### 3.6 Inclusion and exclusion criteria

- Patients and companions who did not wish to participate in the study or refused to continue the study were excluded.
- Patients in the pediatric, neonatal, and outpatient wards; those hospitalized for less than 48 h; and those with a low percentage of alertness for answering the questions were excluded from the study.

## 4 Results

A) Determine criteria and sub criteria of performance assessment and design research model

**Figure 1:**
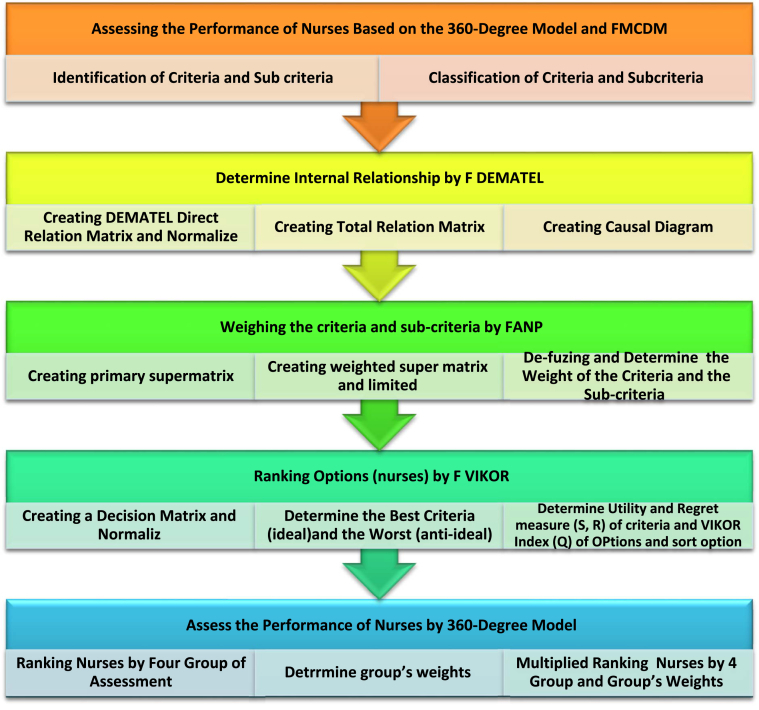
Concept model.

According to library studies, the performance assessment criteria of nurses were classified into five groups. These criteria include technical skills, human skills, and perceived skills (Katz skills); individual characteristics; and compliance with the organization's rules and regulations, which is show in Table 2 (see Figure 1).

**Table 2:** Performance Assessment criteria and sub-criteria.

criteria	sub-criteria	abbr
Personality Features (C1)	Honesty	C_11_
	Control of emotions	C_12_
	Interested and Compassionate	C_13_
	Patience	C_14_
	Prefer organizational interests to individual	C_15_
Human Skills (C2)	Suitable relationships with patients	C_21_
	Training patients	C_22_
	Observing the privacy of patients	C_23_
	Respectful behavior with colleagues	C_24_
	Partnership with colleagues	C_25_
Conceptual Skills (C3)	Make decisions in ambiguous space	C31
	Adaption to changes	C_32_
	Identify weaknesses and strengths	C_33_
	Creativity and innovation	C_34_
Technical Skills (C4)	work with medical equipment	C_41_
	Perform medical procedures correctly	C_42_
	Documentation of patient records	C_43_
Rules and Regulations (C5)	Introducing the patient to an alternative nurse when leaving the ward	C_51_
	Wear uniform properly	C_52_
	Attention to patient safety	C_53_
	Regular patient visits	C_54_

**Figure 2:**
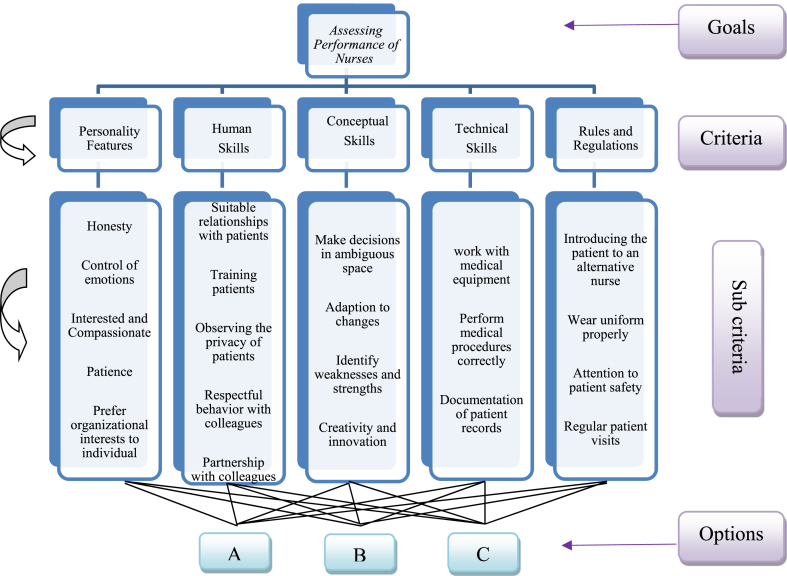
Research model.

Based on the criteria and sub-criteria, the research model, as shown in Figure 2, was designed.

B) Weighing the criteria and sub-criteria
1) Examine the Internal Relationship of Criteria and Sub-Criteria Using the FDEMATEL Method

To examine the internal relations of the criteria and sub-criteria, eight experts were asked to make paired comparisons among the criteria and the sub-criteria. Experts' judgments were based on linguistic options and fuzzy positive numbers (Table 1). The collective direct relation matrix was created by averaging the opinions of the experts. Therefore, the fuzzy direct relation matrix was formed for the criteria and sub-criteria, shown in Tables 3 and 4.

**Table 3:** Fuzzy direct relation matrix for the criteria.

	C1			C2			C3			C4			C5		
	I	M	L	I	M	L	I	M	L	I	M	L	I	M	L
C1	0	0	0	0.42	0.67	0.92	0.08	0.33	0.58	0.08	0.17	0.42	0.17	0.42	0.67
C2	0.25	0.5	0.75	0	0	0	0.25	0.5	0.75	0.17	0.25	0.5	0	0.17	0.42
C3	0	0.25	0.5	0.08	0.33	0.58	0	0	0	0.08	0.17	0.42	0	0.17	0.42
C4	0	0.08	0.33	0.17	0.25	0.5	0.17	0.42	0.67	0	0	0	0	0.08	0.33
C5	0.17	0.33	0.58	0.17	0.42	0.67	0.08	0.17	0.42	0	0.08	0.33	0	0	0

**Table 4:** Fuzzy direct relation matrix for the sub-criteria.

	C_11_	C_12_	C_13_	C_14_	C_15_	C_21_	C_22_	C_23_	C_24_	C_25_	C_31_	C_32_	C_33_	C_34_	C_41_	C_42_	C_43_	C_51_	C_52_	C_53_	C_54_
C_11_	0	0.08	0.42	0.17	0.67	0.33	0.25	0.25	0.17	0.25	0	0	0	0	0	0	0.25	0.08	0.08	0.08	0
C_12_	0	0	0.08	0.5	0.08	0.25	0.08	0	0.42	0.08	0.33	0.25	0.25	0	0.17	0.08	0.08	0	0	0.08	0
C_13_	0.67	0.17	0	0.25	0.83	0.75	0.58	0.58	0.67	0.67	0	0.08	0.08	0	0.08	0.17	0.25	0.58	0.58	0.67	0.7
C_14_	0.08	0.92	0.08	0	0.17	0.5	0.33	0.25	0.5	0.33	0.17	0.17	0.08	0	0.08	0.17	0.17	0.33	0.33	0.33	0.1
C_15_	0.33	0.08	0.58	0.08	0	0.67	0.58	0.67	0.58	0.67	0	0.08	0.17	0	0.08	0.17	0.17	0.67	0.67	0.67	0.8
C_21_	0.25	0.33	0.5	0.5	0.58	0	0.58	0.42	0.08	0.08	0	0.08	0.08	0	0.08	0.17	0.25	0.33	0.33	0.67	0.7
C_22_	0.08	0.17	0.42	0.08	0.58	0.42	0	0.08	0	0	0	0	0.08	0	0	0.08	0.08	0	0	0.08	0.2
C_23_	0	0.17	0.17	0.08	0.5	0.5	0.08	0	0	0	0	0	0	0	0	0.08	0.08	0	0	0.08	0
C_24_	0.17	0.42	0.08	0.33	0.5	0.08	0.08	0.08	0	0.25	0	0.08	0.08	0	0.08	0	0	0.33	0.33	0	0
C_25_	0.5	0.25	0.5	0.5	0.67	0.25	0.25	0.08	0.42	0	0.25	0.42	0.5	0	0.08	0.08	0.08	0.33	0.33	0.08	0
C_31_	0	0.17	0	0.08	0.08	0	0	0	0	0.08	0	0.08	0.58	0	0.08	0.08	0	0	0	0	0
C_32_	0	0.25	0.08	0.5	0.17	0.08	0.08	0	0	0.08	0.17	0	0.08	0	0.08	0.08	0	0	0	0.08	0
C_33_	0.08	0.08	0.08	0.08	0.17	0.08	0.08	0.08	0.08	0.17	0.58	0.33	0	0	0.08	0.08	0	0	0	0	0
C_34_	0	0.17	0.17	0.08	0.17	0.08	0.08	0	0	0.08	0.67	0.25	0.5	0	0.08	0.08	0.08	0	0	0	0
C_41_	0	0.08	0.08	0.08	0.08	0	0	0	0	0.08	0.17	0.17	0.42	0	0	0.08	0	0	0	0.08	0
C_42_	0	0.08	0.33	0.25	0.33	0.17	0.08	0.08	0.08	0.08	0.08	0.08	0.17	0	0.08	0	0.17	0.08	0.08	0.17	0
C_43_	0.42	0.08	0.67	0.08	0.67	0.08	0.08	0.08	0	0.08	0.08	0	0.17	0	0	0.17	0	0	0	0.17	0
C_51_	0.25	0.17	0.33	0.33	0.5	0	0	0	0.33	0.17	0	0	0	0	0	0	0.08	0	0	0.33	0.3
C_52_	0	0.08	0.08	0	0.25	0.17	0.08	0.08	0	0.08	0	0	0	0	0	0	0	0.08	0.08	0.25	0
C_53_	0	0.08	0.67	0.33	0.67	0.33	0.17	0.08	0	0.08	0.08	0	0.25	0	0.08	0.17	0.17	0.42	0.42	0	0.6
C_54_	0.08	0.08	0.67	0.17	0.67	0.5	0.25	0.17	0.08	0.17	0.08	0	0.25	0	0.08	0.17	0.17	0.25	0.25	0.42	0

In the following, the normalized matrix of fuzzy direct relations is formed, and then the total relation matrix (T) is obtained. To normalize the direct relation matrix, we use N = X/K formula. To calculate k, the sum of all rows and columns is computed. K. represents the largest number. All matrix numbers divide into K. To calculate the total relation matrix, 1) Formation of the single matrix, 2) Minus the standard matrix and inverse the matrix, 3) Multiplication of inverse matrices in the normal matrix. The following formula obtains the normalized matrix:

$$3a.\;X=K*A\;\;3b.\;K=\frac{1}{max1\leq i\leq n\sum_{j}^{n}aij}\text{i},\text{j}=1,2,\ldots .\text{n}$$

Total matrix is calculated using the following formula:

$${3\text{c}.\;T=X\left(1-X\right)}^{-1}$$

Finally, the sum of the rows and columns of the matrix (T) are calculated for the criteria and sub-criteria and, as vectors $\tilde{\text{R}}$ (effective) and $\tilde{\text{D}}$ (impressionable), which are given in Table 5.

**Table 5:** Amounts of $\tilde{R}$, $\tilde{D}$, $\tilde{R}+\tilde{D}$, $\tilde{R}-\tilde{D}$

Criteria/Sub criteria	$\tilde{D}$	$\tilde{R}$	$\tilde{R}-\tilde{D}$	$\tilde{R}+\tilde{D}$
Personality Features (C1)	2.75	3.52	0.77	6.26
Honesty	1.01	1.064	0.055	2.07
Control of emotions	1.008	0.897	-0.11	1.90
Interested and Compassionate	1.642	2.211	0.569	3.85
Patience	1.245	1.317	0.072	2.56
Prefer organizational interests to individual	2.17	2.119	-0.05	4.29
Human Skills (C2)	3.53	3.16	-0.367	6.69
Suitable relationships with patients	1.538	1.663	0.125	3.20
Training patients	1.218	0.928	-0.29	2.15
Observing the privacy of patients	1.083	0.786	-0.3	1.87
Respectful behavior with colleagues	1.186	0.907	-0.28	2.09
Partnership with colleagues	1.17	1.54	0.37	2.71
Conceptual Skills (C3)	3.15	2.15	-1.004	5.3
Make decisions in ambiguous space	0.856	0.693	-0.16	1.55
Adaption to changes	0.741	0.724	-0.02	1.47
Identify weaknesses and strengths	1.067	0.811	-0.26	1.88
Creativity and innovation	0.794	0.835	0.041	1.63
Technical Skills (C4)	2.06	2.1	0.044	4.16
work with medical equipment	0.581	0.639	0.058	1.22
Perform medical procedures correctly	0.905	0.92	0.015	1.82
Documentation of patient records	0.795	1.099	0.304	1.89
Rules and Regulations (C5)	1.53	2.59	1.057	4.12
Introducing the patient to an alternative nurse when leaving the ward	1.207	1.048	-0.16	2.25
Wear uniform properly	0.769	0.604	-0.16	1.37
Attention to patient safety	1.371	1.395	0.024	2.77
Regular patient visits	1.262	1.418	0.156	2.68

According to Table 5, criteria with positive $\tilde{\text{D}}$-$\tilde{\text{R}}$, are effective (causal) and criteria with negative $\tilde{\text{D}}$-$\tilde{\text{R}}$, are impressionable (impacted). Among the criteria "Rules and Regulations" are the most effective, and "Conceptual Skills" are the most susceptible. Similarity can be stated for the sub-criteria. DE fuzzy relations matrix among criteria and sub-criteria is shown in Tables 6 and 7.

2) Weighing criteria by fuzzy ANP method

**Table 6:** Defuzzy relations matrix among criteria.

		C1	C2	C3	C4	C5
C1	Personality Features	0.576	1.028	0.788	0.527	0.598
C2	Human Skills	0.727	0.644	0.807	0.53	0.456
C3	Conceptual Skills	0.452	0.575	0.399	0.37	0.352
C4	Technical Skills	0.393	0.56	0.582	0.26	0.306
C5	Rules and Regulations	0.6	0.724	0.576	0.369	0.318

**Table 7:** DE fuzzy Relations Matrix Among Sub-Criteria.

	C_11_	C_12_	C_13_	C_14_	C_15_	C_21_	C_22_	C_23_	C_24_	C_25_	C_31_	C_32_	C_33_	C_34_	C_41_	C_42_	C_43_	C_51_	C_52_	C_53_	C_54_
C_11_	0.03	0.03	0.09	0.05	0.13	0.08	0.07	0.06	0.06	0.06	0.03	0.03	0.03	0.03	0.02	0.03	0.05	0.05	0.03	0.05	0.05
C_12_	0.03	0.03	0.04	0.08	0.05	0.05	0.04	0.03	0.07	0.04	0.06	0.05	0.06	0.03	0.04	0.04	0.03	0.03	0.02	0.04	0.03
C_13_	0.13	0.07	0.1	0.1	0.22	0.17	0.14	0.13	0.14	0.14	0.04	0.05	0.06	0.04	0.04	0.07	0.07	0.14	0.06	0.15	0.15
C_14_	0.04	0.14	0.06	0.04	0.08	0.1	0.08	0.06	0.09	0.07	0.04	0.04	0.05	0.03	0.03	0.06	0.05	0.08	0.03	0.08	0.05
C_15_	0.09	0.06	0.15	0.07	0.12	0.16	0.13	0.13	0.12	0.14	0.04	0.04	0.07	0.04	0.04	0.07	0.06	0.14	0.12	0.15	0.17
C_21_	0.06	0.08	0.13	0.1	0.16	0.07	0.12	0.09	0.06	0.06	0.04	0.04	0.05	0.04	0.04	0.07	0.06	0.09	0.05	0.14	0.14
C_22_	0.04	0.04	0.08	0.04	0.12	0.08	0.03	0.04	0.04	0.04	0.02	0.02	0.03	0.02	0.02	0.04	0.03	0.04	0.03	0.05	0.05
C_23_	0.03	0.04	0.05	0.04	0.1	0.09	0.04	0.03	0.03	0.03	0.02	0.02	0.03	0.02	0.02	0.04	0.03	0.03	0.03	0.04	0.04
C_24_	0.05	0.07	0.05	0.06	0.1	0.05	0.04	0.04	0.03	0.06	0.03	0.03	0.04	0.02	0.03	0.03	0.03	0.07	0.03	0.04	0.04
C_25_	0.1	0.07	0.11	0.1	0.15	0.09	0.08	0.06	0.1	0.05	0.06	0.07	0.09	0.06	0.03	0.05	0.04	0.08	0.04	0.06	0.06
C_31_	0.02	0.03	0.03	0.03	0.04	0.03	0.03	0.02	0.02	0.03	0.02	0.03	0.09	0.09	0.02	0.03	0.02	0.02	0.02	0.03	0.02
C_32_	0.02	0.05	0.04	0.08	0.05	0.04	0.03	0.03	0.03	0.03	0.04	0.02	0.03	0.03	0.03	0.03	0.02	0.03	0.02	0.04	0.03
C_33_	0.03	0.03	0.04	0.04	0.06	0.04	0.03	0.03	0.03	0.05	0.09	0.05	0.03	0.07	0.03	0.03	0.02	0.03	0.02	0.03	0.03
C_34_	0.03	0.04	0.05	0.04	0.06	0.04	0.04	0.03	0.03	0.04	0.1	0.05	0.08	0.02	0.03	0.03	0.03	0.03	0.02	0.03	0.03
C_41_	0.02	0.02	0.03	0.03	0.04	0.03	0.03	0.02	0.02	0.03	0.04	0.03	0.06	0.05	0.01	0.03	0.02	0.02	0.02	0.03	0.02
C_42_	0.03	0.03	0.08	0.06	0.09	0.06	0.04	0.04	0.04	0.04	0.03	0.03	0.04	0.05	0.03	0.02	0.04	0.04	0.03	0.06	0.04
C_43_	0.08	0.03	0.12	0.04	0.14	0.06	0.05	0.05	0.04	0.05	0.03	0.03	0.04	0.03	0.02	0.05	0.02	0.05	0.04	0.07	0.05
C_51_	0.05	0.04	0.08	0.07	0.11	0.05	0.04	0.04	0.07	0.06	0.03	0.03	0.03	0.03	0.02	0.03	0.03	0.04	0.06	0.08	0.07
C_52_	0.02	0.02	0.04	0.03	0.06	0.04	0.03	0.03	0.03	0.03	0.02	0.02	0.02	0.02	0.02	0.02	0.02	0.03	0.02	0.05	0.03
C_53_	0.05	0.04	0.14	0.07	0.15	0.09	0.06	0.06	0.05	0.06	0.04	0.03	0.06	0.04	0.03	0.06	0.05	0.09	0.04	0.06	0.12
C_54_	0.05	0.04	0.14	0.07	0.15	0.11	0.08	0.07	0.06	0.07	0.04	0.03	0.06	0.04	0.03	0.06	0.05	0.08	0.04	0.1	0.06

In the next step, based on the calculations of the FDEMATEL method, weighing the criteria and the sub-criteria was done as follows.

- Creating primary supermatrix: Using pairwise weighting, the primary supermatrix was formed.
- Creating a weighted supermatrix: After creating the supermatrix, the weighted supermatrix was formed.
- Creating a limited supermatrix: The weighted supermatrix was converged and created a limited supermatrix.

Finally, by De fuzzing the weights by the center of gravity method, the weight of the criteria and the sub-criteria is determined, which show in Table 8 (see Table 9).

**Table 8:** Weight of the criteria and sub-criteria.

Criteria (Weight and Rank)			Abbr	Sub-Criteria	Weight and Rank		Weight and Finally Rank	
Personality Features (C1)	0.195	(3)	C_11_	Honesty	0.024	(5)	0.005	11
			C_12_	Control of emotions	0.031	(4)	0.006	10
			C_13_	Interested and Compassionate	0.043	(2)	0.008	8
			C_14_	Patience	0.034	(3)	0.007	9
			C_15_	Prefer organizational interests to individual	0.062	(1)	0.012	6
Human Skills (C2)	0.273	(1)	C_21_	Suitable relationships with patients	0.067	(1)	0.018	2
			C_22_	Training patients	0.049	(4)	0.013	5
			C_23_	Observing the privacy of patients	0.043	(5)	0.012	6
			C_24_	Respectful behavior with colleagues	0.052	(3)	0.014	4
			C_25_	Partnership with colleagues	0.061	(2)	0.017	3
Conceptual Skills (C3)	0.241	(2)	C_31_	Make decisions in ambiguous space	0.054	(2)	0.013	5
			C_32_	Adaption to changes	0.046	(4)	0.011	7
			C_33_	Identify weaknesses and strengths	0.089	(1)	0.021	1
			C_34_	Creativity and innovation	0.054	(3)	0.013	5
Technical Skills (C4)	0.166	(4)	C_41_	work with medical equipment	0.034	(3)	0.006	10
			C_42_	Perform medical procedures correctly	0.068	(1)	0.011	7
			C_43_	Documentation of patient records	0.064	(2)	0.011	7
Rules and Regulations (C5)	0.125	(5)	C_51_	Introducing the patient to an alternative nurse when leaving the ward	0.034	(2)	0.004	12
			C_52_	Wear uniform properly	0.02	(4)	0.003	13
			C_53_	Attention to patient safety	0.038	(4)	0.005	11
			C_54_	Regular patient visits	0.033	(3)	0.004	13

**Table 9:** The best criteria (ideal) and the worst (anti-ideal).

Quality of criteria	the best criteria (ideal)	the worst criteria (anti-ideal)
Positive Criteria	$f_{j}^{*}$ = Max f_j_	$f_{j}^{-}$ = Min f_j_
Negative Criteria	$f_{j}^{-}$ = Min f_j_	$f_{j}^{*}$ = Max f_j_

As Table 5 shows, the highest weight is on the “identify the strengths and weaknesses” criterion that gained priority. The “Suitable relationships with patients” criterion gained the second priority, and the “partnership with colleagues” was the third priority among the 21 criteria.

C) Ranking Options by Fuzzy VIKOR

After determining the weight of criteria and sub-criteria by F. DANP method, Fuzzy VIKOR method was used to rank the options (nurses).

In this stage, supervisors used the VIKOR questionnaire to assess the performance of nurses. Scoring was done with the paired comparison matrix and based on linguistic judgment and fuzzy positive numbers (Table 2). The ranking steps are as follows:

1. Creating a decision matrix: The rating matrix of options based on criteria was formed.

$$3\text{d}.\;x={\left[x_{ij}\right]}_{\text{m*n}}=\begin{array}{c} C_{1}\\ A1\\ A2\\ .\\ .\\ .\\ Am \end{array}\left[\begin{array}{cccccc} C_{2} & . & . & . & . & C_{n}\\ x11 & x12 & . & . & . & x1n\\ x21 & x22 & . & . & . & x2n\\ . & . & . & . & . & .\\ . & . & . & . & . & .\\ . & . & . & . & . & .\\ xm1 & xm2 & . & . & . & xmn \end{array}\right]$$

2. Unscaling the decision matrix is done by linear normalization.

$$3\text{e}.\;n_{ij}=\frac{x_{ij}}{\sqrt{\sum_{i=1}^{m}x_{ij}^{2}}}$$

3. Determining the best criteria (ideal) and the worst (anti-ideal): The best and the worst values were determined for each sub-criteria.
4. Determine Utility and Regret measure ($\tilde{\text{R}}and\tilde{\text{S}}$) of criteria

Utility expresses the relative distance of the i-option from the ideal point.

$$3\text{f.}\;{\text{S}}_{\text{i}}=\sum_{i=1}^{n}W_{j}\frac{f_{j}^{*}f_{ij}}{f_{j}^{*}f_{j}^{-}}$$

Regret expresses the maximum discomfort of the i-option in avoiding the ideal point.

$$3g.\;{\text{R}}_{\text{i}}=Max\left[W_{j}\frac{f_{j}^{*}f_{ij}}{f_{j}^{*}f_{j}^{-}}\right]$$

5. Determine VIKOR Index (Q) of Options

$$3\text{h.}Q_{i}=v\left[\frac{S_{i-}S^{*}}{S^{-}-S^{*}}\right]+\left(1-v\right)\left[\frac{R-R^{*}}{R^{-}-R^{*}}\right]\;\text{\{}S^{*}\text{:}\min S_{i}\;{\text{R}}^{*}\text{:}\min{\text{R}}_{i}\;{\text{S}}^{-}\text{: Max}S_{i}\;R^{-}\text{:Max}R_{i}\text{\}}$$

The agreement of the decision group determines the parameter v. If v > 0.5; then there is a lot of agreement. If v < 0.5, then there is a little agreement.

6. Sort Options based on $\tilde{\text{R}}and\tilde{\text{S}}$*And Q ~*

Options are sorted small to large in three groups according to Q, R, and S amounts. The best option is a case that has the smallest Q amount.

According to Table 10, the results are as follows:

3i. D7 <D8 <D4 <D5 D1 <D6 <D3 <D2

**Table 10:** The amounts of Q, R, and S.

de fuzzy						
$\tilde{\text{Q}}$		$\tilde{\text{R}}$		$\tilde{\text{S}}$		Rank
V = 0.5						
0.0011	D7	0.0174	D7	0.0027	D7	1
0.0296	D8	0.0208	D4	0.0103	D8	2
0.0432	D4	0.0208	D8	0.0439	D4	3
0.0665	D5	0.0226	D5	0.0675	D5	4
0.123	D1	0.0291	D1	0.0876	D1	5
0.1473	D6	0.0301	D6	0.13	D6	6
0.1679	D3	0.0307	D3	0.1691	D3	7
0.2154	D2	0.0358	D2	0.1924	D2	8

As far as the supervisors were concerned, nurse D7 got the best ranking (a nurse in the dialysis ward).

To achieve the highest ranking, we must pay attention to two conditions:

First condition: Acceptable advantage

3j. (Q 2) − (Q 1)≥ 1/ (n-1)→0/03-0/001≥0/143

Since the first conditionwas not accepted, the second condition was examined.

3k.(Q 3) − (Q 1)< 1/ (n-1)→0/043-0/00 < 0/143

As seen, the second condition was established. Therefore, nurse D7 was acknowledged as a qualified nurse in the dialysis ward.

D) Assessing the Performance of Nurses Based on 360-Degree Model

Other assessment groups included co-workers, patients, and their companions and self-assessment ranked by the nurses themselves using the fuzzy VIKOR method. The ranking results is shown in Table 11.

**Table 11:** Ranking results of 4 group performance assessment.

	Rank	Name		Rank	Name		Rank	Name		Rank	Name
Supervisors Assessment	1	Nurse 7	Partners Assessment	1	Nurse 7	Clients Assessment	1	Nurse 7	Self-assessment	1	Nurse 8
	2	Nurse 8		2	Nurse 4		2	Nurse 5		2	Nurse 5
	3	Nurse 4		3	Nurse 8		3	Nurse 8		3	Nurse 7
	4	Nurse 5		4	Nurse 5		4	Nurse 4		4	Nurse 4
	5	Nurse 1		5	Nurse 3		5	Nurse 3		5	Nurse 1
	6	Nurse 6		6	Nurse 6		6	Nurse 2		6	Nurse 6
	7	Nurse 3		7	Nurse 2		7	Nurse 1		7	Nurse 3
	8	Nurse 2		8	Nurse 1		8	Nurse 6		8	Nurse 2

To calculate the final score of performance assessment in the 360-degree model, different assessments were summarized according to their weights. So separate pairwise comparisons were done for assessment groups.

As shown in Table 12, each group's weights were different. The supervisor's assessment score was 0.521, with the highest weight, and the self-assessment was 0.042 with the lowest weight.

**Table 12:** Weight of four groups of nurses' performance Assessment.

Assessment groups	Weight
Supervisors	0.521
Coworkers	0.198
Patients and their companions	0.239
Self-assessment	0.042

In the following, the ranking results of nurses by four groups and group's weights multiplied and determined results ranking, which is shown in Table 13.

**Table 13:** The result of nursing ranking in the dialysis ward.

Weight of 4 groups	Supervisors	partners	Clients	Self-assessment	Final Ranking
Name of Nurses	0.521	0.198	0.198	0.042	
Nurse 7	1	1	1	3	0.27
Nurse 8	2	3	3	1	0.60
Nurse 4	3	2	4	4	0.77
Nurse 5	4	4	2	2	0.86
Nurse 1	5	8	7	5	1.52
Nurse 3	7	5	5	7	1.53
Nurse 6	6	6	8	6	1.62
Nurse 2	8	7	6	8	1.83

The final ranking of nurses in a different ward of the hospital is shown in Table 14.

**Table 14:** The result of nursing ranking in All Wards.

Rank	abbr	Name	Ward
1	N7	Nurse 3	Children and Infants
2	N8	Nurse 6	CCU
3	N4	Nurse 4	Dialysis
4	N5	Nurse 5	Emergency Department
5	N1	Nurse 12	Post CCU
6	N6	Nurse 2	surgery
7	N3	Nurse 7	Internal

## 5 Discussion

In this study, we used the 360-Degree Model and DEMATEL, ANP, and VIKOR combination approach in the fuzzy environment to assess and select qualified nurses. We identified criteria in five dimensions and then assigned 21 sub-criteria for them. To follow, criteria and sub-criteria were weighted by the DEMATEL and ANP method in the fuzzy environment and the arrangement of the importance of criteria was: human skills (0.273), conceptual skills (0.241), technical skills (0.166), personality features (0.195), and rules and regulations (0.125). Also, with ANP method assessment of the four groups was weighted, and the order of importance was: supervisors (0.521), patients and their companions (0.239), coworkers (0.198), and self-assessment (0.042). Finally, we used the VIKOR questionnaire in the fuzzy environment to assess the performance of nurses by four groups.

Azar and Sepehrirad (2010) used the fuzzy AHP technique and 360-degree method to assess employees' performance in four dimensions and then assigned 19 sub-criteria for them. Afterwards criteria and sub-criteria were weighted by the AHP method, the arrangement of the importance of criteria was: technical skills (0.419), perceptual skills (0.301), human skills (0.224), and personal characteristics (0.051). Also with the AHP method four groups' assessment was weighted, in which the arrangement of importance was: supervisors (0.502), coworkers (0.335), subordinates (0.106), self-assessment (0.054). Finally, the final scores of the employees' performance were calculated by applying a mathematical model of integration [37]. The main advantage of the DEMATEL and ANP method was in solving problems with complex relationships. We determined the internal relationship of criteria and also sub-criteria by this method to determine the cause-effect relationship among criteria and also among sub-criteria. This method helps us to have a better understanding of the relationships among criteria and sub-criteria and express our views more powerfully. Whilst the AHP method is opposed to ANP, one-way relationships among criteria are considered.

Also, the VIKOR method is suitable for decision making on issues with inappropriate criteria (different measurement units) and which are conflicting. VIKOR method selects the best option and brings it as close as possible to the ideal option. Similar studies which used this method are; Chiu, Tzeng, &Li, (2013) who used DANP with VIKOR to improve e-store business. They used DEMATEL based Analytic Network Process (DANP) for the substantial weighting of criteria and used the VIKOR method for ranking and selection of the best option with different criteria [38]. Yang, Shieh, & Tzeng (2013) used the VIKOR technique based on DEMATEL and ANP for information security risk control assessment. This study used the VIKOR for ranking the information-security-risk-control objectives and control areas and for improvement of the normalization process in ANP and constructed the interrelations among criteria by using DEMATEL [39]. Alimohamadiyan & shafiee (2016) for performance assessment and improving the gaps between teaching hospitals, used DEMATEL, TOPSIS, ANP, and VIKOR. They used DEMATEL to examine the interrelationships among criteria and create a network relations map and then used the ANP to determine the importance (weight) of the criteria. To improve the gap of each criterion for achieving the goals TOPSIS was employed, and for comparing and ranking of hospitals VIKOR was used [40]. In some studies like Azar and Sepehrirad (2010) AHP technique was employed. Mombini (2016) used the DEMATEL-AHP-VIKOR combination method to prioritize private-sector investment strategies that proposed the AHP model provides an easy math form instead of complex forms such as ANP. In this study, due to the interdependence of criteria such as profit, opportunities, costs, and threats the DEMATEL method was used to determine the relative importance of the criteria and VIKOR was also employed as a powerful method for ranking options [41].

## 6 Conclusion

In this research, we tried to provide a new method for assessing the performance of nurses in a hospital by identifying the criteria and subcriteria and in turn select qualified nurses in the hospital. The DEMATEL and ANP methods were used to discover the weighted criteria, and subcriteria. Then, qualified nurses were assessed and selected by VIKOR questionnaires and the 360-degree model. The advantage of the proposed method is more realistic results than other methods because the criteria and sub-criteria are weighted, and the importance of each is determined. Furthermore, the results of this study can be used to assess the performance of other medical groups in hospitals.

## 7 Suggestions

Nurses are the largest care providers in hospitals, and attention to their performance can play a significant role in improving the quality of services. By assessing their performance periodically by different groups and with various criteria, their strengths could be identified and enhanced. Furthermore, encouraging, reprimanding, or conducting training courses are recommended to promote nurses' performance, thereby improving the quality of their services, which leads to positive steps in the health system.

## Declarations

### Author contribution statement

M, Rahati: Conceived and designed the experiments; analyzed and interpreted the data.

N. Rohollahi, Z. Sakeni: Contributed reagents, materials, analysis tools or data; Performed the experiments.

H. Zahed, R. Nanakar: Wrote the paper; Analyzed and interpreted the data.

### Funding statement

This work was supported by Kashan University of Medical Sciences, Iran (Project Number: 93161).

### Competing interest statement

The authors declare no conflict of interest.

### Additional information

No additional information is available for this paper.
